# Twisted MoSe_2_ Homobilayer Behaving as a *Heterobilayer*

**DOI:** 10.1021/acs.nanolett.4c01764

**Published:** 2024-07-23

**Authors:** Arka Karmakar, Abdullah Al-Mahboob, Natalia Zawadzka, Mateusz Raczyński, Weiguang Yang, Mehdi Arfaoui, Gayatri, Julia Kucharek, Jerzy T. Sadowski, Hyeon Suk Shin, Adam Babiński, Wojciech Pacuski, Tomasz Kazimierczuk, Maciej R. Molas

**Affiliations:** † Institute of Experimental Physics, Faculty of Physics, University of Warsaw, Pasteura 5, 02-093 Warsaw, Poland; ‡ Center for Functional Nanomaterials, Brookhaven National Laboratory, Upton, New York 11973, United States; § Department of Chemistry, Ulsan National Institute of Science and Technology, Ulsan 44919, Republic of Korea; ∥ Département de Physique, Faculté des Sciences de Tunis, Université Tunis El Manar, Campus Universitaire, 1060 Tunis, Tunisia; ⊥ Center for 2D Quantum Heterostructures, Institute for Basic Science (IBS), Suwon 16419, Republic of Korea; # Department of Energy Science, Sungkyunkwan University, Suwon 16419, Republic of Korea

**Keywords:** Twisted heterostructure, TMDC, MoSe_2_, homobilayer, excitons, energy transfer

## Abstract

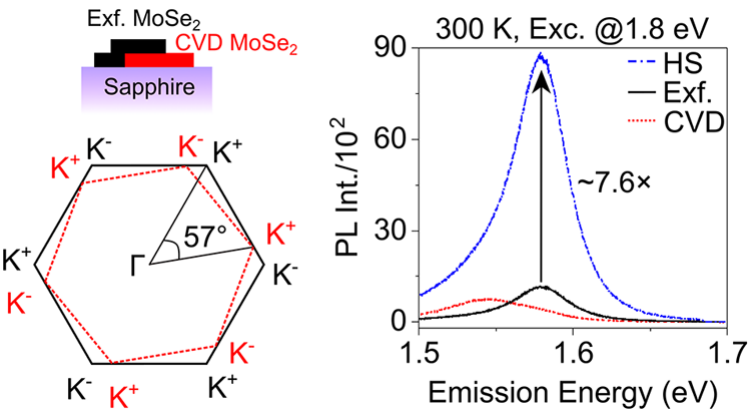

Heterostructures (HSs) formed by the transition-metal dichalcogenide materials have shown great promise in next-generation (opto)electronic applications. An artificially twisted HS allows us to manipulate the optical and electronic properties. In this work, we introduce the understanding of the energy transfer (ET) process governed by the dipolar interaction in a twisted molybdenum diselenide (MoSe_2_) homobilayer *without* any charge-blocking interlayer. We fabricated an unconventional homobilayer (i.e., HS) with a large twist angle (∼57°) by combining the chemical vapor deposition (CVD) and mechanical exfoliation (Exf.) techniques to fully exploit the lattice parameter mismatch and indirect/direct (CVD/Exf.) bandgap nature. These effectively weaken the interlayer charge transfer and allow the ET to control the carrier recombination channels. Our experimental and theoretical results explain a massive HS photoluminescence enhancement due to an efficient ET process. This work shows that the electronically decoupled MoSe_2_ homobilayer is coupled by the ET process, mimicking a “true” heterobilayer nature.

Heterostructures (HSs) made by the vertical stacking of monolayers (1Ls) of two-dimensional (2D) transition-metal dichalcogenides (TMDCs) have been a trending topic^1^ over the past decade due to their potential usage in the next generation ultrathin optoelectronic and photonic device applications.^2,3^ Twisting the materials while forming the HS creates Moiré superlattices, which then modulate the electronic and optical properties.^4,5^ Twisted 2D HSs have been extensively studied to understand the various Moiré physics, such as flat bands,^6^ structure of Moiré exciton,^7^ and inter/intralayer excitonic modulations,^8,9^ just to name a few. Competing interlayer charge^10,11^ (CT) and energy transfer^12−14^ (ET) are the two main photocarrier relaxation routes in the HSs formed by the semiconducting TMDCs. Thus, understanding the competing interlayer processes and creating a comprehensive understanding are essential for the realization of TMDC-based optoelectronic applications. In simple words, CT happens when carriers tunnel from the higher-to-lower-lying energy states, whereas the ET due to the dipole–dipole interaction is analogically similar to the two pendulums with identical oscillating frequencies in classical mechanics. A few recent studies^15−18^ have already explored the dependence of the twist angle on the interlayer CT process in 2D HSs. Nonetheless, up until now, there has been no clear understanding of the complex ET process in twisted TMDC HSs including the homobilayers. An earlier report^19^ has shown an ET process in a homobilayer of tungsten disulfide (WS_2_) separated by a thin hexagonal boron nitride (hBN) interlayer to block the indirect optical transition. Until now, an experimental study on the ET process in a twisted homobilayer *without* any charge-blocking interlayer, i.e., in atomically close proximity, has yet to be reported, since the traditional interlayer ET rate (∼1 ps)^12^ in TMDCs is found to happen at a much slower rate than the CT process (<50 fs).^10,18^ Thus, CT is considered to be the dominating mechanism in 2D HSs within nanometer separation.

In this work, we explore an unusual ET coupling in a twisted molybdenum diselenide (MoSe_2_) homobilayer made by the combination of a chemical vapor deposition (CVD) and mechanical exfoliation (hereafter Exf.) technique. Instead of choosing the conventional HS fabrication routes, such as direct CVD growth^20,21^ or polymer-based dry transfer methods,^22^ we chose an unorthodox method of combining the CVD and Exf. technique. Such a mixed approach allows us to exploit the different lattice parameters between the two materials produced by different growth techniques, create a homobilayer (*i.e.*, HS) with a clean large-area interface, and take advantage of the direct/indirect (Exf./CVD) bandgaps overlap. Until now, this type of homobilayer with a twist angle has never been studied. We intentionally opt for the twisted HS with a large rotation (57°) combined with the lattice mismatch to minimize the charge tunneling^15,23^ mediated by the excitonic wave functions overlapping without any charge-blocking interlayer.^14^ It is also important to mention that being an optically “bright” material 1L MoSe_2_ photoluminescence (PL) intensity significantly quenches at room temperature as compared to the cryogenic temperature.^24^ This study reveals a massive amount of Exf. PL enhancement at room temperature from the HS area by a factor of ∼7.6× due to an efficient interlayer ET process from the CVD to Exf. layer. We employ a series of optical and electron spectroscopy techniques at low (5 K) and room temperature (300 K), such as optical second harmonic generation (SHG), differential reflection contrast (RC), photoluminescence excitation (PLE), circularly polarized PL, time-resolved PL (TR-PL), reflection high-energy electron diffraction (RHEED), and μ-beam low energy electron diffraction (μLEED). The experimental results, complemented by the density functional theory (DFT) calculations, help us to demonstrate that the homobilayer effectively behaves as a “heterobilayer”; *i.e.*, each layer works independently, resulting in no PL quenching effect due to the evolution of indirect bandgap as per the conventional twisted homobilayer structure.^8,25^

We grow isolated 1L MoSe_2_ triangles on the ultraflat sapphire substrate using the CVD method. We then deterministically transfer the top Exf. MoSe_2_ layer to create a HS (see Experimental Details for the sample fabrication procedure). Figure 1a shows the optical micrograph, while the inset shows the schematic illustration of the sample’s cross-section. The optical SHG technique has been proven to be a nondestructive method to determine the stacking angle in the artificial twisted 2D HSs.^26^ Our SHG experiment (see Experimental Details) shows an ∼57° twist angle between the two layers (Figure 1b). Figure 1c shows a graphic illustration of the 57° rotational angle between the hexagonal Brillouin Zones (BZs) of two 1Ls.

**Figure 1:**
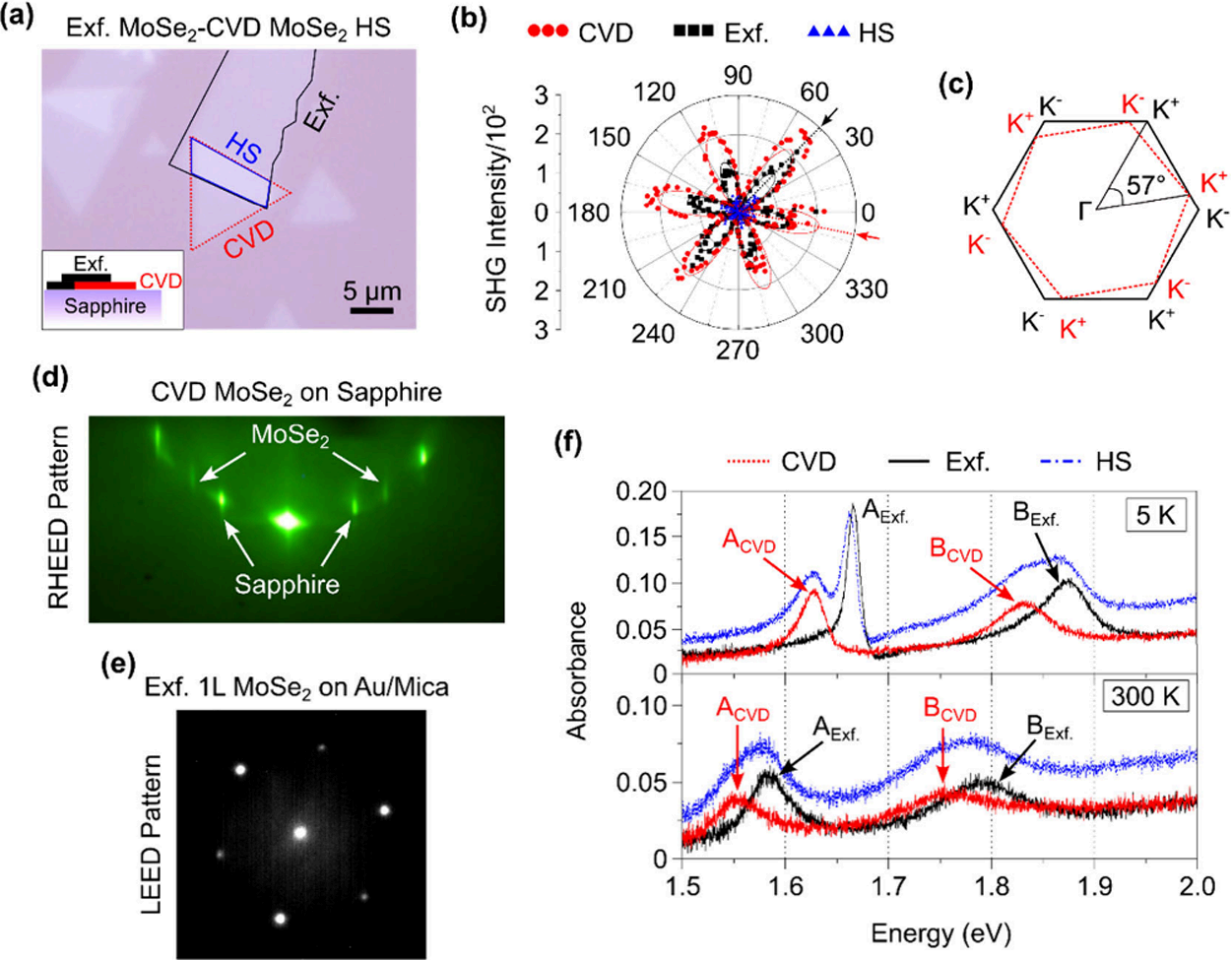
Material characterization. (a) Optical image of the twisted Exfoliated (Exf.) MoSe_2_-chemical vapor deposition (CVD) MoSe_2_ homobilayer (HS) on the ultraflat sapphire substrate. Inset is the schematic illustration of the side-view of the sample. (b) Optical second harmonic generation (SHG) intensity polar plot to identify the rotational angle between the individual layer (1Ls). SHG intensities were divided by a factor of 10^2^. The angle difference between the two main lobes (pointed by arrows) of the CVD and Exf. Layers are found to be ∼57°. (c) Schematic illustration of the 57° twisted Brillouin Zones (BZs) of the two layers as determined from the SHG measurements. (d, e) Reflection high-energy electron diffraction (RHEED) patterns from the CVD layer on sapphire and μLEED pattern of 1L Exf. Flake on the Au/Mica substrate, respectively. We find that there is an ∼2.8% lattice parameter mismatch between the two materials. (f) Absorbance spectra converted from the differential reflection contrast (RC) measurements taken at cryogenic temperature (5 K) and room temperature (300 K), top and bottom panel, respectively. CVD excitonic peak positions show a constant red-shift as compared to the Exf. peak at both the temperature regime. HS peaks show summation of peaks from both the layers.

RHEED is an ideal tool to compare the reciprocal lattice parameters in thin films.^27^ From the RHEED measurements (more information in the Experimental Details), we find the CVD MoSe_2_ lattice parameter to be ∼3.25 Å (Figure 1d). Due to the different substrate condition and lack of reference point, we do not consider the Exf. MoSe_2_ RHEED pattern on the SiO_2_/Si substrate (Figure S1) for our analysis. Rather, we perform a μLEED measurement (Figure 1e) to find the true lattice constant of ∼3.34 Å from the Exf. flake (more in the Experimental Details). The CVD growth^28^ on the sapphire substrate could cause this strain-induced lattice mismatch as compared to the relaxed (Exf.) top layer. Later, we show how this ∼2.8% lattice mismatch combined with ∼57° twist angle between the two layers led us to observe an unprecedented ET process in the HS area.

The absorbance (Abs.) spectra (Figure 1f) were calculated from the differential RC measurements (see Experimental Details) using the following equation:^29^
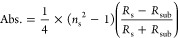
where *n*_s_ = 1.77 is the refractive index of the underlying sapphire substrate. The Abs. spectra show an overall red-shift in the CVD peak positions as compared to the Exf. peaks at both temperature regimes (5 and 300 K). The HS data show a combination of peaks from both the layers. At 5 K, a slight red-shift in the Exf. A peak from the HS area as compared to the isolated Exf. A peak could be a result of a change in the dielectric environment.

PLE provides material’s quasi-absorption spectra and is an ideal tool for studying the interlayer ET process.^12,14^ The low temperature (5 K) μ-PLE measurements (see Experimental Details) show a well separated narrow neutral (*X*_o_) and bound excitonic emission (*X*^–^) from the individual Exf. and HS area, and the CVD PL emission shows a typical^30^ pronounced emission centered ∼1.55 eV (Figure 2a–c). We also see a phonon crossover^31^ with the PL emission at near resonant A excitation in the Exf. PLE map (indicated by the white arrows in Figure 2b). Upon increasing the temperature to 300 K, all of the peaks merged to an expected broad emission feature (Figure 2d–f). The excitation and emission ranges in all the PLE measurements were kept constant to compare any temperature-induced shifts or changes in the spectra. 5 K PL emission at an excitation of ∼1.88 eV, matching the Exf. MoSe_2_ B excitonic level (along the horizontal solid line in Figure 2a–c), shows a slight enhancement of the *X*_o_ emission by a factor of ∼1.2× in the HS area as compared to the individual Exf. area (Figure 2g). At low temperature, the *X*^–^ emission from the HS area was quenched as compared to the Exf. spectrum (Figure 2g), which may be a result of the PL filtering effect^32^ due to the transfer of the bound states from the Exf. to CVD layer. Moreover, the reduced bound-state emission proves good coupling between the individual layers. The room temperature (300 K) PL emission at an excitation energy matches the Exf. MoSe_2_ B level (∼1.8 eV), showing a massive increase of the HS PL emission by a factor of ∼7.6× (Figure 2h). The PL enhancement factor is defined as the ratio of the maximum integrated PL intensity from the HS area to the isolated Exf. emission, while all of the experimental parameters remain the same. The PLE plot, which is basically a change of the emission landscape as a function of the excitation energies (along the vertical dotted line in Figure 2b,c), shows a similar slight enhancement of the HS emission at 5 K (Figure 2i) and an overall massive enhancement at 300 K (Figure 2j) as compared to the Exf. emission. We would like to emphasize the fact that, in our PL measurements, we do not observe any indirect emission due to the emergence of an indirect bandgap at the *K*–Γ transition as per the traditional TMDC homobilayer studies.^8,25^

**Figure 2:**
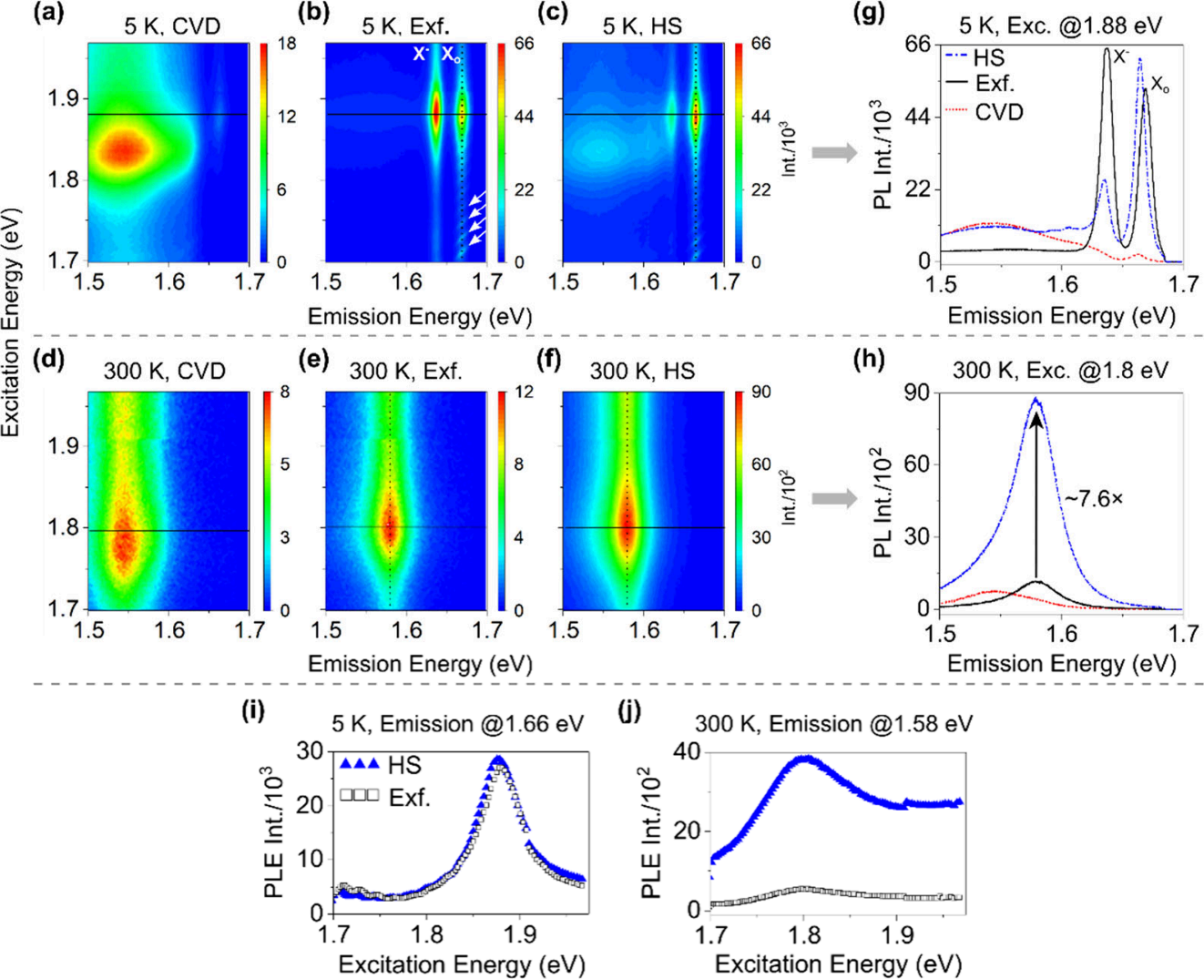
PLE measurements. (a–c) Low temperature (5 K) photoluminescence excitation (PLE) measurements taken from the CVD, Exf., and HS areas, respectively. Intensities were divided by a factor of 10^3^. In Figure (b) the white arrows indicate the phonon lines crossing the PL emission. (d–f) Room temperature (300 K) PLE measurements from the CVD, Exf,. and HS areas, respectively. Intensities were divided by a factor of 10^2^. (g) 5 K photoluminescence (PL) emission plot at 1.88 eV excitation energy, *i.e.*, along the horizontal solid line in (a–c). HS neutral exciton peak emission (*X*_o_) shows an enhancement of ∼1.2× as compared to the Exf. neutral exciton peak emission. (h) 300 K PL emission plot at 1.8 eV excitation energy, *i.e.*, along the horizontal solid line in (d–f). HS neutral exciton peak emission shows an enhancement of ∼7.6× as compared to the Exf. neutral exciton peak emission. (i) 5 K PLE plot from the Exf. and HS areas at 1.66 eV emission energy (along the vertical dotted line in (b,c)). HS emission shows a slight PL increment as compared to the Exf. layer throughout the entire excitation range. (j) 300 K PLE plot from the Exf. and HS areas at 1.58 eV emission energy (along the vertical dotted line in (e, f). HS emission shows a massive PL increment as compared to the Exf. layer throughout the entire excitation range.

To verify the data reproducibility, we prepare two more HSs with different large twist angles, and both show a pronounced HS PL emission at room temperature (Supporting Information Figures S3–S4). Furthermore, we perform a room temperature PL intensity map at a resonant excitation of Exf. MoSe_2_ B level (Figure S5) to confirm the uniformity of the pronounced PL emission throughout the entire HS area. These results show that the reported PL enhancement is not related to a sample-specific condition but due to a very unusual coupling between the two layers, as discussed in the later sections. It is important to mention that we only consider the Exf. PL enhancement in the HS area is due to an ET process from the CVD layer, as the HS PL emission shape, line width, and position perfectly match with the individual Exf. area (Figure S6). Also, ET from the CVD to Exf. layer happens only to the *X*_o_ level. Because, *X*^–^ emission is related to the local effect/intrinsic doping in the sample, while ET is mainly associated with the coupling between the nonlocalized (free) excitons. Thus, our main focus is to study the enhanced *X*_o_ HS PL emission due to ET from the CVD to the Exf. layer.

Circularly polarized light creates an exciton (e–h pair) in a single valley based on the optical selection rule. These photocarriers can promote the intervalley scattering between the *K*^*+*^ and *K*^*–*^ valleys *via* different scattering mechanisms, mainly governed by the Coulomb interactions^33^ or phonon-mediated scattering.^34^ Thus, studying the circularly polarized PL emission and measuring the degree of circular polarization (*P*) provide us useful information about the valley population.^34^*P* is determined by the following equation:
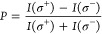
where *I*(σ^+^/σ^–^) are the right and left circularly polarized PL intensities, respectively. Circularly polarized excitation matching with the material’s B excitonic level results in PL emission from the material’s A excitonic level from the same valley, conserving the photocarriers’ total spin state (Figure 3a). Low temperature (5 K) circularly polarized PL emission at an excitation energy of 1.88 eV (a technical discussion is provided in the Experimental Details) shows a low-to-high change of the *P* value for the neutral (*X*_o_) emission from the CVD (55%), Exf. (76%), and HS (92%) areas, respectively (Figure 3b–d). Upon increasing the temperature to 300 K, the CVD area sees an increase (89%) and the Exf. area experiences a reduction (50%) in *P* (Figure 3e,f), whereas the HS area demonstrates an unchanged *P* value of 92% (Figure 3g). An almost constant PL polarization with the increasing temperature was observed earlier.^35^ However, the large circular polarization value of 92% from the HS area is to our knowledge the highest number reported to date from a mono- or bilayer TMDC system at room temperature.^36−38^ A high *P* value at room temperature is a necessary condition for the future valleytronics applications based on the optically initialized *K*-valley polarization.^35^ The high *P* value from the HS area indicates that the initially injected polarization primarily contributes to the PL emission.

**Figure 3:**
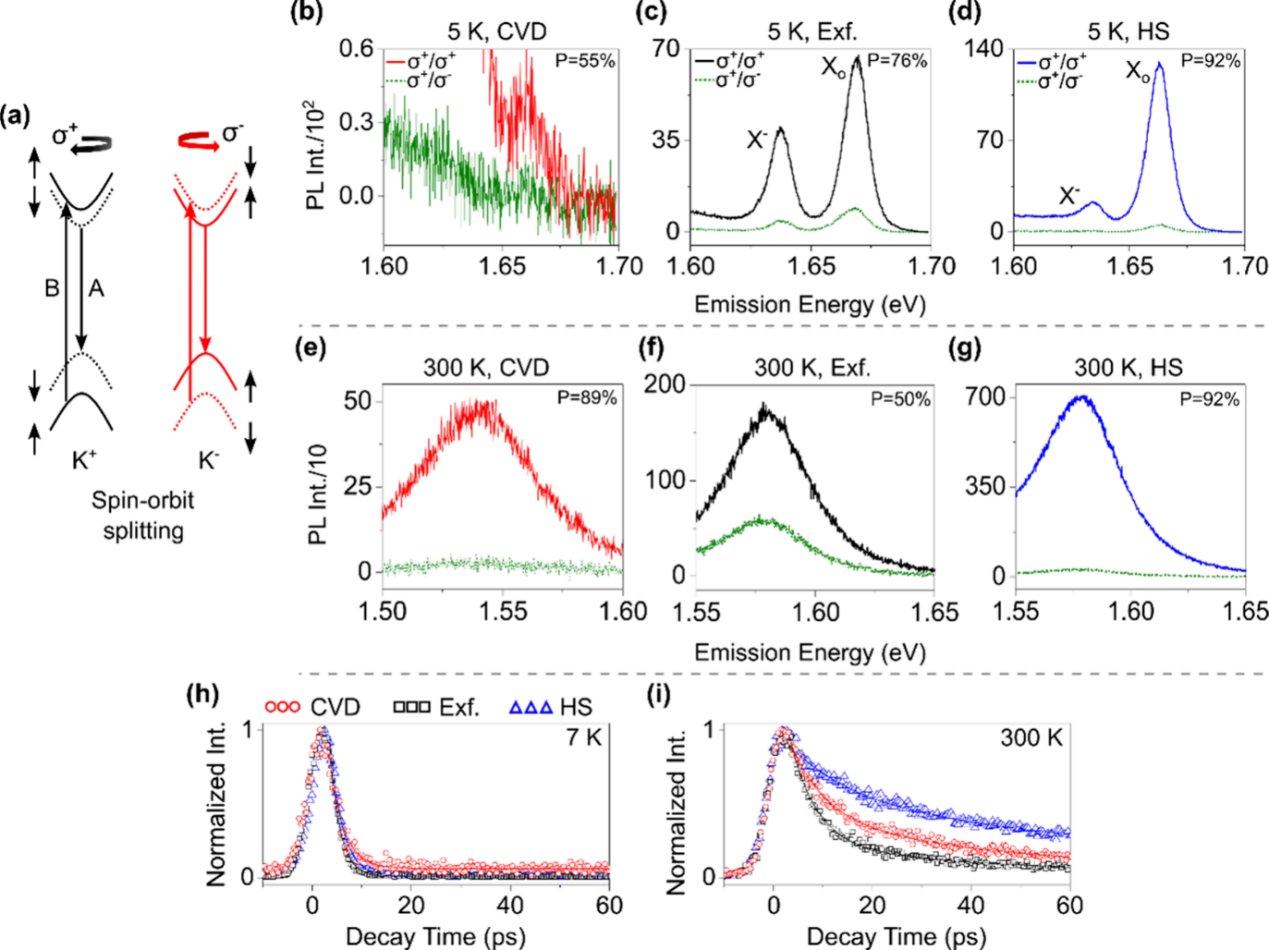
Circularly polarized PL and TR-PL. (a) Schematic illustration of the circularly polarized PL emission at a resonant excitation of Exf. MoSe_2_ B level. Valley-selective right (σ^+^) and left (σ^–^) circularly polarized excitations/emissions are marked with the black and red lines, respectively. (b–d) 5 K circularly polarized PL emission at an excitation of 1.88 eV from the CVD, Exf., and HS areas, respectively. In each of the layers, the PL comparisons were made with the same excitation/detection (σ^+^/σ^+^) and opposite excitation/detection (σ^+^/σ^–^) configuration to obtain the degree of circular polarization (*P*) of the neutral exciton (*X*_o_). PL intensities were divided by a factor of 10^2^. (e–g) Similar circularly polarized PL emission at 300 k with 1.88 eV excitation of CVD, Exf., and HS regions, respectively. PL intensities were divided by a factor of 10. (h, i) Time resolved PL (TR-PL) spectra from the three regions measured at 7 and 300 K, respectively. At 7 K, the decay times of the *X*_o_ emissions from the CVD, Exf., and HS areas are ∼2.87 ps, ∼ 2.08 ps, and ∼2.50 ps, respectively. At 300 K, the spectra were fitted using a biexponential decay function to obtain the fast (τ_1_) and slower (τ_2_) time constants. τ_1_ and τ_2_ values from the CVD, Exf., and HS areas are ∼7.26 ps, ∼55.00 ps; ∼5.39 ps, ∼49.15 ps; and ∼12.94 ps, ∼96.80 ps, respectively. The hollow symbols represent the real signal and the solid lines are the fitted data.

The PL lifetimes (*i.e.*, neutral excitonic decay time) determined by the TR-PL spectra (more details in the Experimental Details and Supporting Information) show the usual behavior from the CVD and Exf. areas but a completely surprising characteristic from the HS area at 300 K (Table 1 and Figure 3h,i). At 7 K, the Exf. decay time undergoes a slight reduction as compared to the CVD PL lifetime, and the HS PL lifetime fell in between the two layers. Both the CVD and Exf. decay times at 300 K show a higher value due to the increased phonon-assisted intervalley scattering.^23,39^ However, a prolonged HS decay time at room temperature, even more than the individual layers, suggests a different mechanism, as discussed in the next section. The bound-state *X*^–^ emissions from all the three regions show a longer decay time at low temperature as compared to the *X*_o_ emission (Figure S7). Since the ET process happens faster than the thermalization of exciton distribution, the excess carrier relaxation due to the ET will dominate only by the radiative recombination of the neutral (intrinsic) exciton. Thus, this bound-state prolonged emission will be suppressed, and as a result, we see a quenching of the HS *X*^–^ emission in the PLE measurement (Figure 2g).

**Table 1:** TR-PL Decay Time Obtained from the Curve Fitting as Described in the Experimental Details

	7 K	300 K	
	Decay Time (ps)	τ_1_ (ps)	τ_2_ (ps)
CVD	2.87 ± 0.11	7.26 ± 0.36	55.00 ± 1.91
Exf.	2.08 ± 0.03	5.39 ± 0.16	49.15 ± 2.10
HS	2.50 ± 0.03	12.94 ± 1.1	96.80 ± 5.00

The DFT calculations (details are given in the Supporting Information) reveal an indirect gap for the CVD MoSe_2_ with the energetically lowest *K*–*Q* transition and a direct bandgap for the Exf. MoSe_2_ layer is located at the *K* points (Figure 4a,b). The indirect bandgap of the CVD MoSe_2_ is expected due to the smaller lattice parameter (∼3.25 Å) compared to the relaxed (Exf.) MoSe_2_ layer (∼3.34 Å), mimicking a similar scenario under a compressive strain.^40,41^ By comparing the band structures with the calculated work function (WF), we see that the two layers form an “almost” type-II HS (Figure 4c). Now, after knowing the band alignment, we take into consideration all the possible scenarios to explain our experimental results step-by-step as follows:

I. The red-shift in the CVD excitonic level as compared to the Exf. layer (Figure 1f) is due to the similar effect in increased excitonic binding energy upon applying a compressive strain.^41^
II. The large twist angle combined with the lattice mismatch effectively weakens the interlayer charge tunneling.^15,23^
III. As shown in Figure 4d, upon resonant B excitation electrons populate in the CVD conduction band (CB) *K* valley (step 1), following by an immediate scattering to the *Q* valley (step 2). These electrons then scatter back to the *K* valley *via* e-phonon scattering (step 3)^142^ before transferring to the Exf. layer *via* dipolar coupling (step 4) and then radiatively recombine to the ground state (step 5) creating excess Exf. PL emissions from the HS area.
IV. At 5 K, the kinetic energy  (*K*_B_ is the Boltzmann constant) of the electron is negligible, <1 meV. This small electron *E*_K_ combined with the low phonon population make the *Q* → *K* scattering (step 3) less probable at low temperature. On the other hand, at 300 K, the higher electron *E*_K_ ∼ 40 meV, paired with the increased optical-phonon scattering,^23^ make the *Q* → *K* scattering (step 3) more effective, thus allowing an efficient ET coupling at room temperature. This scheme explains both the slight HS PL enhancement at 5 K and the massive PL growth at 300 K (Figure 2i,j).
V. The CVD band structure also helps us to interpret the increase in the CVD *P* value at 300 K (89%) as compared to the 5 K (55%), which is due to the surge in the intervalley scattering (*K* ↔ *Q*).
VI. Another interesting point is that, at 300 K, the prolonged HS TR-PL lifetime (Table 1) combined with the band alignment (Figure 4c) indicates a both-way excitonic exchange (possibly multiple times) *via* the ET process before radiatively recombining at the Exf. layer. Moreover, the excitonic upconversion due to the WF mismatch (∼120 meV) between the layers can be more probable when the large momentum phonon population is high, *i.e.*, at room temperature.^42^

**Figure 4:**
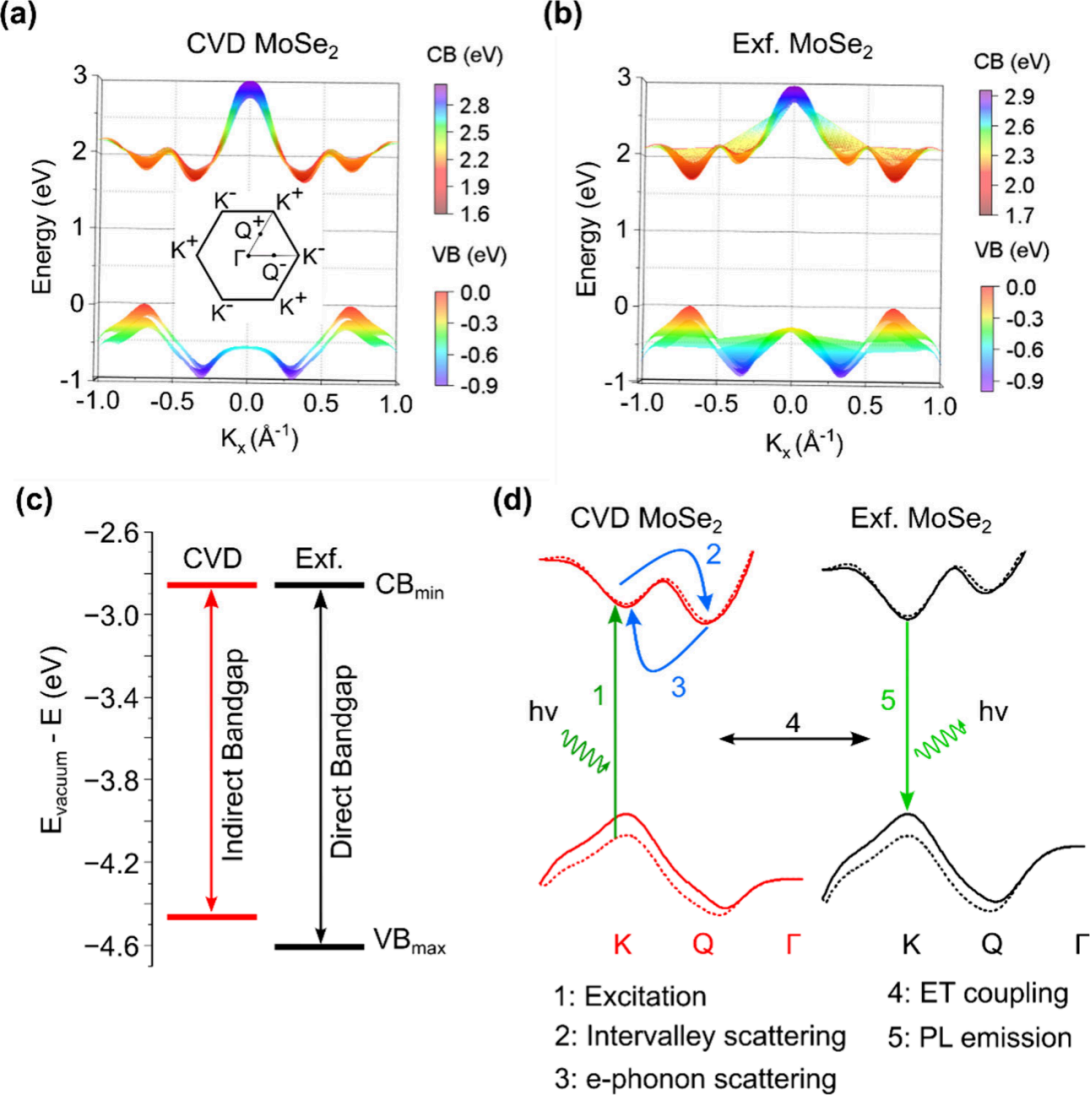
Band alignment and photocarrier relaxation pathways. (a, b) Density functional theory (DFT) computed 3D energy band diagram of CVD and Exf. layers, respectively. The DFT computed energy bandgaps were matched with the energy levels obtained from the absorbance spectra. The CVD layer shows an indirect bandgap at *K*–*Q* transition, whereas the Exf. layer shows a direct bandgap at the *K*–*K* transition. Inset of (a) shows the cut in the BZ along which the computed band structures were plotted. (c) Schematic illustration of the band alignment between the two layers matching the calculated work function (WF) indicates an “almost” type-II HS configuration. (d) Graphical representation of the photocarrier relaxation pathways in the HS at resonant B excitation. The spin–orbit splitting band structures are represented by the solid and dotted lines. For a simplified view, step-by-step transitions are labeled in the diagram.

Also, it is worth mentioning that close to AB (also called 2*H*) stacking helped us achieve very strong interlayer coupling^8^ in the HS area. Isolating the short-range interlayer CT and Dexter-type ET due to the electron wave function overlap is beyond the scope of this present work.

In conclusion, we experimentally show that the electronically decoupled large twisted MoSe_2_ homobilayer is effectively coupled *via* the interlayer ET process mediated by e-phonon interactions, which imitates a *heterobilayer* structure. This ET results in an ∼7.6× PL enhancement from the HS area at room temperature. In such a HS, the large twist angle, together with the ∼2.8% lattice mismatch, effectively weakens the interlayer CT process, allowing the ET to take over the control of the photocarrier relaxation pathways *without* any charge-blocking interlayer. The DFT calculations prove an “almost” type-II band alignment. This then explains the atypical prolonged excitonic lifetime from the HS area obtained at room temperature. Hence, we show that the MoSe_2_ homobilayer behaves like a “heterobilayer”. We also expect that the other Mo-based TMDC homobilayers (MoS_2_ and MoTe_2_) will have an effect similar to that reported in the present work.

Our previous^13,14^ and present works already showed that, whenever there is an energetically resonant overlap between the optically excited states, ET dominates over the interlayer CT processes. Considering the fast interlayer CT time scale (<50 fs),^10,18^ we can expect a faster ET period. However, finding the real ET rate requires a separate ultrafast study. Nonradiative ET in TMDC HSs is less explored as compared to the CT process due to the difficulties in direct observation of the dipole–dipole coupling, even with modern state-of-the-art spectroscopy/microscopy techniques. In the past few years, the rapid advancement of the time- and angle-resolved photoemission spectroscopy (TR-ARPES) technique has already enabled the direct observation of the rapid formation of the exciton,^43,44^ measurement of the excitonic wave function,^45^ structure/formation of the Moiré exciton,^7,46^ and interlayer CT process^47^ in semiconducting TMDC materials. We also believe that in the upcoming years the time- and energy-resolution of the state-of-the-art TR-ARPES will be greatly enhanced to become an ideal tool to study (visualize) the ET processes in the newly emerging 2D HSs. Another interesting point in our experiment is the difference in the PL enhancement factors between the PLE and circularly polarized PL measurements. This clearly indicates a role of the inter- and intravalley transitions in the ET process, and a detailed study is required to uncover the e-phonon scattering mechanism. We strongly believe that this work will be a stepping stone in understanding the complex ET processes in twisted HSs, which is ideal for designing next-generation optoelectronic, electronic, and photonic applications.

## Experimental Details

### HS Fabrication

The triangular 1L MoSe_2_ flakes on the sapphire substrates were grown using the CVD method as described in ref (28). For the top MoSe_2_ layer, first, we exfoliated MoSe_2_ flakes from the bulk crystal onto the Gel-Pak (PDMS) films. After confirming the 1L nature of the exfoliated MoSe_2_ flakes by the PL measurements, we carefully stacked the 1Ls on top of the CVD flakes using a custom-built transfer setup. The samples were then annealed at 100 °C for ∼30 min under ∼40 mbar (30 Torr) pressure to make good physical contact between the layers. We obtained commercially available bulk MoSe_2_ crystals for exfoliation from the HQ Graphene.

### Characterization

The optical SHG experiment was performed using a Coherent Mira 900 Ti:Sapphire 76 MHz oscillator tuned to 800 nm. The polarization of the incoming fundamental beam was rotated using a half-waveplate placed directly in front of the microscope objective. The resulting 400 nm SHG beam was detected by using a Czerny–Turner spectrometer equipped with a CCD camera. The weak HSs SHG signals from the first and third samples (Figure 1b and Figure S4b) are a clear signature of destructive interferences of SH fields, hence confirming the ∼57° and ∼47° twist angle, respectively.^26^ Whereas, the strong HS SHG signal from the second sample (Figure S3b) shows a constructive interference of SH fields and confirms the ∼31° twist angle. In all the SHG data, the corresponding lobes from the 1L regions are indicated with arrows to determine the twist angles.

RHEED equipment is a part of the Molecular Beam Epitaxy reactor made by SVT Associates, Inc. RHEED patterns were observed on the CVD MoSe_2_ sample grown on the sapphire substrate, whereas for the Exf. MoSe_2_ RHEED pattern we cleaved the commercially available bulk MoSe_2_ crystals directly on the SiO_2_/Si substrate to increase the material coverage on the substrate for better RHEED pattern visualization. Background pressure was maintained at ∼10^–9^ Torr. Before the measurement, samples were quickly heated in an ultrahigh vacuum chamber up to 600 °C to degas the surface adsorbents and then immediately dropped down to 200 °C. RHEED patterns were acquired with 8 kV accelerating electron energy. The CVD lattice parameter was obtained at ∼3.25 Å by using the sapphire as a reference.^48^

The Exf. MoSe_2_ sample was further studied employing the low-energy electron microscopy/photoelectron emission microscopy (LEEM/PEEM) technique.^49^ To determine the lattice parameter of 1L Exf. MoSe_2_ using the μLEED pattern, the Exf. flake was transferred onto Au coated mica. The LEED pattern was recorded from a selected 1.5 μ area of the sample (μLEED) in LEEM. For calibrating the μLEED pattern, the Ru(0001)Ox surface was prepared *in situ* in LEEM immediately after taking the MoSe_2_ μLEED data. The MoSe_2_ μLEED pattern was scaled using O(2 × 1,1 × 2)-Ru(0001) spots (Figure S2) with identical optical LEEM setting and alignment. The obtained in-plane lattice parameter of Exf. MoSe_2_ is ∼3.34 Å.

We performed the differential RC measurements using a tungsten–halogen lamp focused by a Mitutoyo Plan Apo SL 50× (N.A. 0.42) objective and directed into a spectrometer. Samples were loaded into a continuous flow cryostat and cooled with liquid helium (LHe). The differential reflectance is defined by (*R*_s_ – *R*_sub_)/(*R*_s_ + *R*_sub_), where *R*_s_ is the reflected light intensity from the MoSe_2_ flakes and *R*_sub_, from the sapphire substrate.

The μ-PLE experiments were performed using a supercontinuum light source coupled with a monochromator as an excitation source. Samples were cooled using the same cryostat used in the RC measurements. The incident light was focused on the samples using a Mitutoyo Plan Apo SL 100× (N.A. 0.55) objective. The average power on the sample was kept only at ∼13 μW (laser spot diameter ∼1 μm) to avoid any high power induced nonlinear effects from the sample. For the circularly polarized PL measurement, we used the same setup at a fixed excitation of ∼1.88 eV photons. λ/4 waveplates were used in the excitation and detection paths to control the left and right circularly polarized light. The λ/4 waveplate at the detection side was rotated to control either the same excitation/detection (σ^+^/σ^+^) or opposite excitation/detection (σ^+^/σ^–^) configuration.

TR-PL measurements were performed using a S20 synchroscan streak camera. In these experiments, the sample was excited with a femtosecond Optical Parametric Oscillator (OPO) with 660 nm light at 7 K and 690 nm light at 300 K to match the Exf. MoSe_2_ B level at a repetition rate of 76 MHz. The sample was cooled with LHe inside a continuous flow cryostat. We kept the average power on the sample at ∼30–40 μW.

## Data Availability

The data that support the findings of this study are openly available at https://doi.org/10.58132/N2AFEB. The technical details of the theoretical simulations are available from the corresponding authors upon reasonable request.

## Supporting Information Available

RHEED pattern of Exf. MoSe_2_, μLEED pattern of RuO_2_, PLE measurements of 31° and 47° twist samples, PL intensity map, normalized PL emission comparison, TR-PL fitting equations and spectra of *X*^–^ emission, and details of the DFT calculations (PDF)
