# Supplementary material for: Twisted MoSe2 Homobilayer Behaving as a Heterobilayer
Source: Nano Lett. 2024 Jul 23;24(31):9459–67. doi: 10.1021/acs.nanolett.4c01764 (PMC11311526; doi:10.1021/acs.nanolett.4c01764)
Supplement: Supplementary file 1 — nl4c01764_si_001.pdf [file nl4c01764_si_001.pdf]

SUPPORTING INFORMATION: Twisted $MoSe_2$ Homobilayer Behaving as a *Heterobilayer*

Arka Karmakar[1*], Abdullah Al-Mahboob[2#], Natalia Zawadzka[1], Mateusz Raczyński[1], Weiguang Yang[3], Mehdi Arfaoui[4], Gayatri[1], Julia Kucharek[1], Jerzy T. Sadowski[2], Hyeon Suk Shin[3,5,6], Adam Babiński[1], Wojciech Pacuski[1], Tomasz Kazimierczuk[1], Maciej R Molas[1†]

[1] Institute of Experimental Physics, Faculty of Physics, University of Warsaw, Pasteura 5, 02-093 Warsaw, Poland

[2] Center for Functional Nanomaterials, Brookhaven National Laboratory, Upton, NY 11973, USA

[3] Department of Chemistry, Ulsan National Institute of Science and Technology, Ulsan 44919, Republic of Korea

[4] Département de Physique, Faculté des Sciences de Tunis, Université Tunis El Manar, Campus Universitaire 1060 Tunis, Tunisia

[5] Center for 2D Quantum Heterostructures, Institute for Basic Science (IBS), Suwon 16419, Republic of Korea

[6] Department of Energy Science, Sungkyunkwan University, Suwon 16419, Republic of Korea

***** arka.karmakar@fuw.edu.pl; karmakararka@gmail.com

[#] aalmahboo@bnl.gov

[†] maciej.molas@fuw.edu.pl

RHEED pattern of Exfoliated (Exf.) $MoSe_2$:

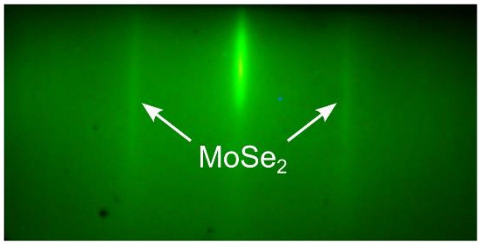

Figure S1: RHEED pattern of the Exf. $MoSe_2$ flakes on SiO_2/Si substrate. Despite the high crystal quality of exfoliated flakes, the observed diffraction lines are broader than in the case of epitaxial CVD flakes (Fig. 1d in the main manuscript), due to the random orientation of the Exf. flakes.

µLEED pattern of O(2x1, 1x2)-Ru(0001):

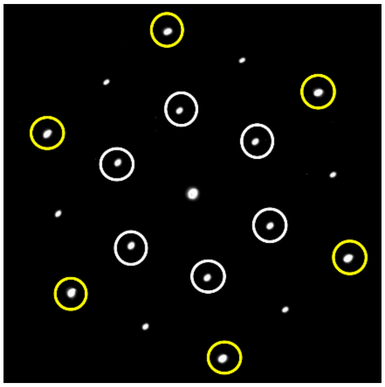

Figure S2: µLEED pattern from the ruthenium-oxide surface taken with 40 eV electron beam. O(2×1,1×2)-Ru(0001) formed by oxidation of Ru(0001) surface. Yellow circles represent the Ru(1×1) spots and white circles represent the O(2×1,1×2) spots.. This surface was used to calibrate the reciprocal space to obtain the Exf. $MoSe_2$ lattice parameter.

PLE measurements on the 2nd HS with ~31° twist angle:

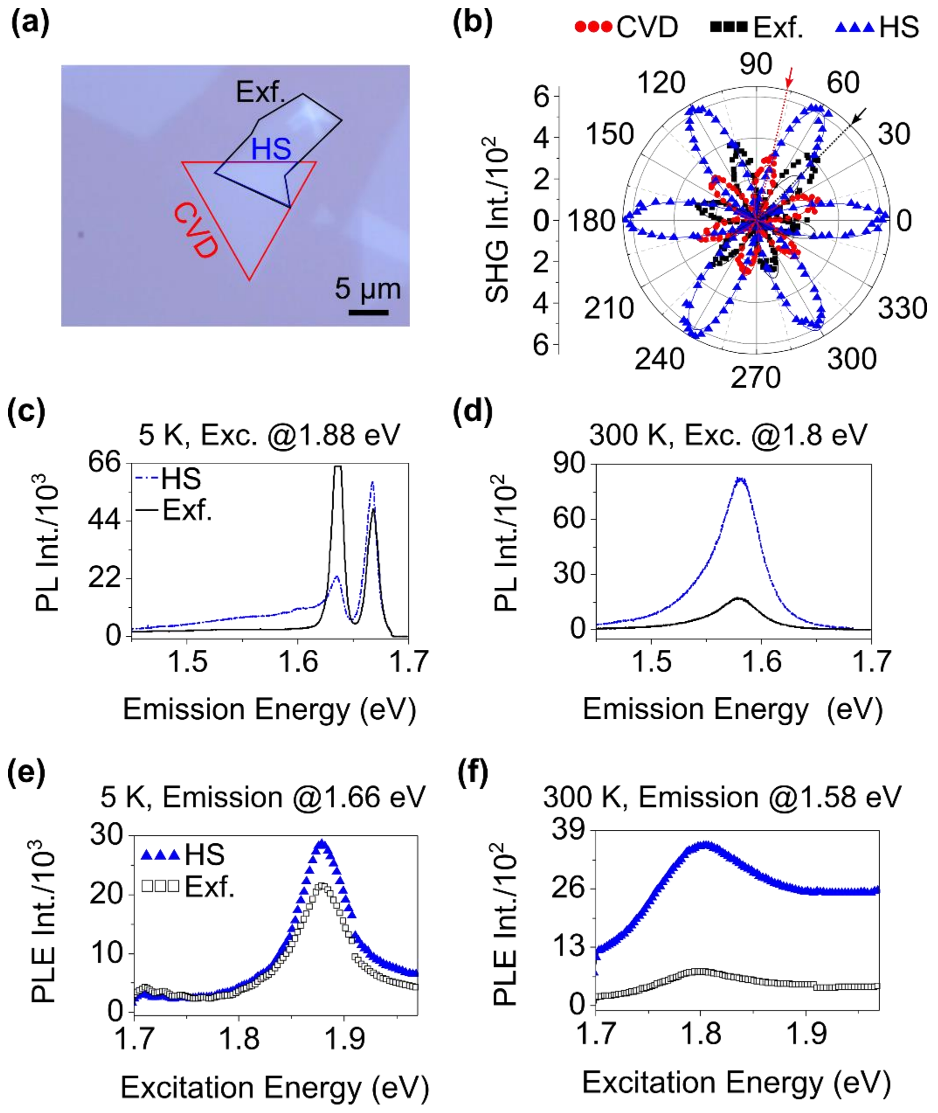

Figure S3: (a) Optical micrograph of the 2nd $MoSe_2$ homobilayer fabricated by the Exf. and CV technique. (b) Optical SHG measurements show ~31° rotation between the two layers. (c)-(d) PL spectra at an excitation matching with the Exf. $MoSe_2$ B level at 5 K and 300 K, respectively. At low temperature the neutral PL emission from the HS area shows a slight enhancement as compared to the Exf. layer. Whereas, at room temperature HS PL shows ~4.8× intensity enhancement. (e)-(f) PLE plots show a similar increase in the PL emission throughout the entire excitation range at 5 K and 300 K, respectively.

PLE measurements on the 3rd HS with ~47° twist angle:

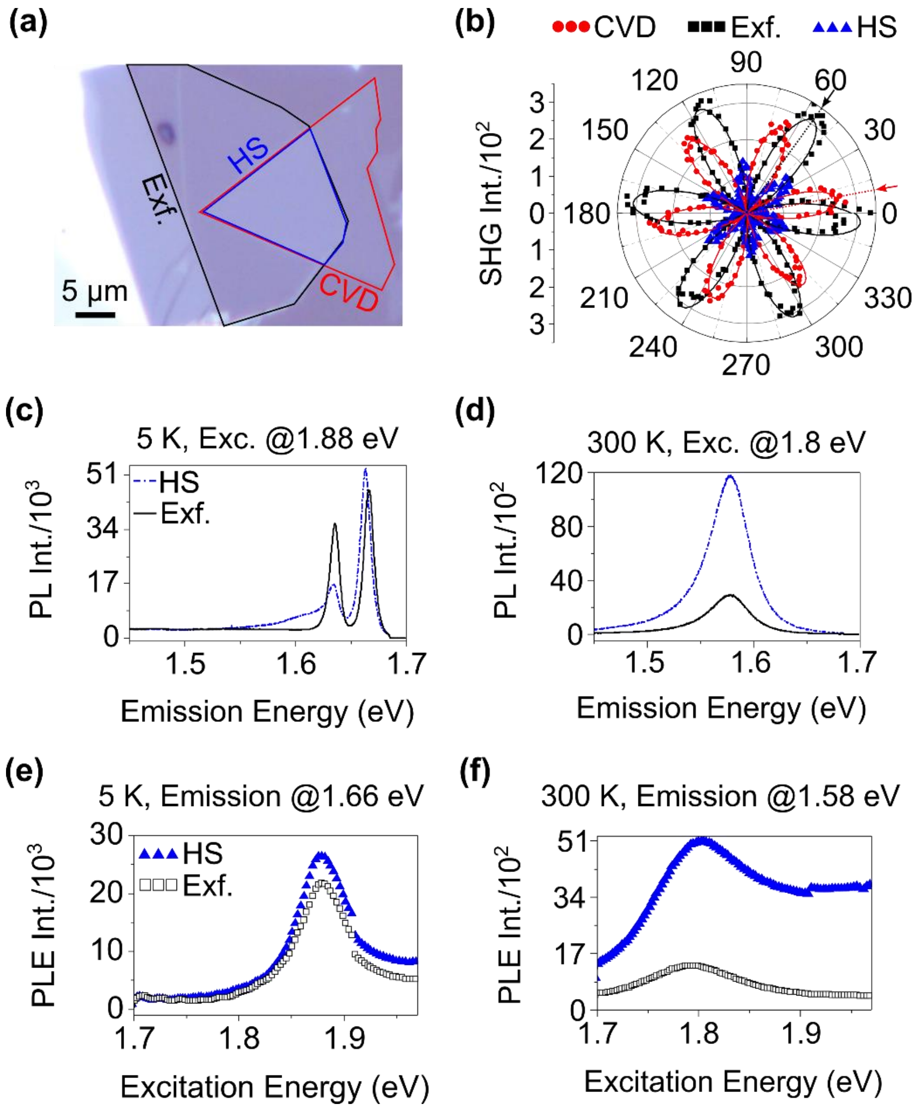

Figure S4: (a) Optical micrograph of the 3rd $MoSe_2$ homobilayer fabricated by the Exf. and CV technique. (b) Optical SHG measurements show ~47° rotation between the two layers. (c)-(d) PL spectra at an excitation matching with the Exf. $MoSe_2$ B level at 5 K and 300 K, respectively. At low temperature the neutral PL emission from the HS area shows a slight enhancement as compared to the Exf. layer. Whereas, at room temperature HS PL shows ~4× intensity enhancement. (e)-(f) PLE plots show a similar increase in the PL emission throughout the entire excitation range at 5 K and 300 K, respectively.

PL intensity map of the 3rd HS with an excitation of 1.8 eV:

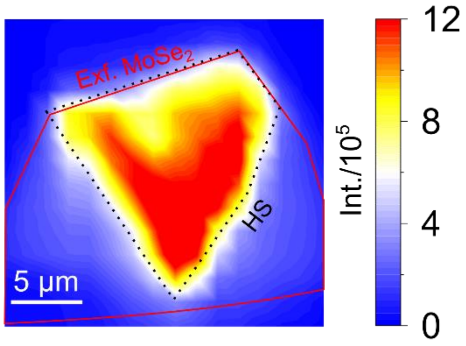

Figure S5: Room temperature PL map of the total integrated intensity from the 3rd sample clearly shows an overall massive enhancement throughout the entire HS area. We note that, there is a slight non-uniformity in the homogeneous intensity distribution, which is typical for an exfoliated flake. However, the PL intensity from the HS is still higher as compared to the surrounding isolated layers.

Normalized PL emission comparison:

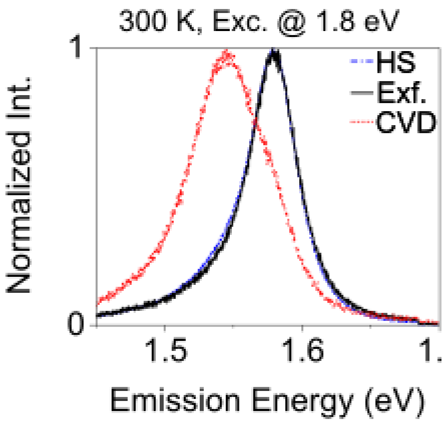

Figure S6: Normalized PL emission comparison under 1.8 eV excitation at room temperature. HS PL peak position, linewidth and shape perfectly matches with the Exf. PL emission.

Fitting equations of the TR-PL spectra:

Low temperature (7 K) spectra were fitted using a mono-exponential decay function:

$$y = y_o + A_1 \exp\left(-\frac{x - x_o}{\tau_1}\right)$$

where, y_o is the y offset, x_o is the x offset, A_1 is the amplitude and τ_1 is the time constant. Whereas, the room temperature (300 K) spectra were fitted using a bi-exponential decay function:

$$y = y_o + A_1 \exp\left(-\frac{x - x_o}{\tau_1}\right) + A_2 \exp\left(-\frac{x - x_o}{\tau_2}\right)$$

τ_1 and τ_2 are the faster and slower time constants, respectively, and A_1, A_2 are the corresponding amplitudes. The TR-PL spectra taken at the both temperature range were fitted after 3 ps from the rise time to exclude the system response and any hot-carriers related effect.

TR-PL spectra of the bound excitonic emission:

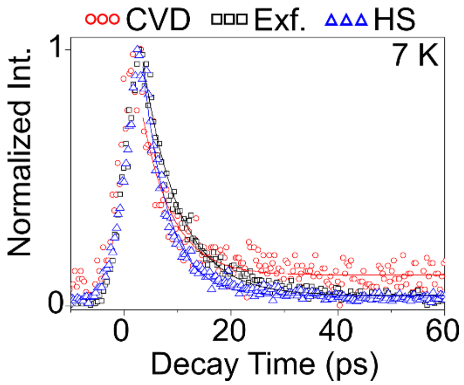

Figure S7: Time resolved PL (TR-PL) spectra of the bound exciton (X^-) emission from the three regions measured at 7 K. The spectra were fitted using a single exponential decay function. The time constants from the CVD, Exf. and HS areas are 5.659 ± 0.369 ps, 6.801 ± 0.147 ps and 4.733 ± 0.096 ps, respectively. The hollow symbols represent the real signal and the solid lines are the fitted data.

Theoretical Calculation:

Band structure calculation: DFT calculations is performed using the Materials Studio CASTEP (CAmbridge Serial Total Energy Package) version 2024, *ab initio* Total Energy Program (first principles methods using CASTEP).[1] We did not develop or validated any DFT codes employed in the calculation. Plane wave basis set cut-off in CASTEP as well as other parameters except K-point mesh were set to ultra-fine default option set in the software package/CASTEP module. Prior to the band structure calculation, we performed the geometry optimization (GO) constraining the lattice parameter obtained from experimental values. To obtain band structure of ML TMD, crystal was cleaved parallel to the layer (c* terminated) and then a vacuum slab > 20 Å was added along the c* to make the 1L TMD structures. In-plane lattice parameter of CVD $MoSe_2$ was fixed at a = 3.25 Å and a = 3.33 Å was considered for the Exf. $MoSe_2$. In DFT calculation, Atomic solver, Dirac (Author Chris J. Pickard, Cambridge University) in CASTEP module is used when requesting spin-orbit coupling and on the fly generated pseudopotentials. Self-consistent calculation performed for the test configuration for Se: $1s^2$ $2s^2$ $2p^6$ $3s^2$ $3p^6$ $3d^{10}$ $4s^2$ $4p^4$ and for Mo: $1s^2$ $2s^2$ $2p^6$ $3s^2$ $3p^6$ $3d^{10}$ $4s^2$ $4p^6$ $4d^5$ $5s^1$. Pseudo atomic calculation performed for Se $3d^{10}$ $4s^2$ 4p4 and for Mo $4s^2$ $4p^6$ $4d^5$ $5s^1$.

Band structure calculation was performed considering the fine k-spacing in single point energy calculation corresponding to 20x20x1 supercell and spectral k-spacing of ~0.006$Å^{-1}$ along M-K-Γ-M. 2D band structure (E vs K_x, K_y) is obtained from DOS.band output file (eigen values vs K_x, K_y) in which k-point mesh in the computation is set corresponding to 100x100x1 supercell with unique 5000 k-points within k-space defined by the reciprocal space a*/2 (0 to a*/2) and b* (-b*/2 to b*/2). For GO and computing the ground state band structure of 1Ls $MoSe_2$, DFT-D (GGA+dispersion correction) method: Perdew-Bruke-Ernzerhof (PBE) GGA functional[2] is employed along with the dispersion correction (van der Waals correction accounted employing the dispersion correction for DFT) by Tkatchenko-Scheffler (TS) method.[3] After computation of the electronic band structure in CASTEP, scissors have applied to the band structure plot to match with the bandgap obtained from the RC spectroscopy measurements.

Work function calculation: The WF calculations are performed based on the DFT by using the plane-wave (PW) method as implemented in QUANTUM ESPRESSO code.[4-6] We use full-relativistic norm-conserving pseudopotentials for electron-ion interaction and PWs with a kinetic energy cutoff of 70 Ry to expand electronic wavefunctions. The interlayer vdW interaction is described by using the DFT-D3 dispersion correction method. The cutoff energy of the PW expansion is optimized to 70 Ry, which ensures the convergence of the system, and a 25x25x1 Γ-centered Monkhorst-Pack k-mesh grid is used in the BZ for structural optimization and performance calculations. The convergence thresholds for energy and atomic forces are fixed at 10^{-4} eV and 10^{-5} eV/Å, respectively. 15 Å vacuum layer along z-axis is added to interrupt the artifacts of the periodic boundary conditions, spin-orbit coupling (SOC) is considered in this work.

The estimated work function (WF) is defined as $W^{CVD/Exf} = E_{Vacuum}^{\mathrm{CVD/Exf}} - E_F^{CVD/Exf}$, where $W^{CVD/Exf}$, $E_F^{CVD/Exf}$ and $E_{Vacuum}^{CVD/Exf}$ denote the WF, Fermi level and vacuum level of CVD/Exf, respectively. The values of $W^{CVD/Exf}$ are estimated in Figure S8, which displays the average electrostatic potential in vacuum for both monolayers (CVD and Exf.). The estimation can be acquired via averaging the total electrostatic potential along the direction out-of-plane. Indeed, From the DFT calculation, the planar-averaged electrostatic potential is given as:

$$V(z) = \frac{1}{A_{xy}} \iint \mathrm{V(x,y,z)dxdy} + \mathrm{E_0}$$

where A_{xy} is the transverse area of the $MoSe_2$ slab. The constant E_0 is an energy shift applied to make $V(z) = 0$ in the vacuum region. The WF of CVD and Exf. 1L $MoSe_2$ are 4.48 eV and 4.60 eV, respectively, with CVD exhibiting a 120 meV lower WF compared to Exf. Indeed, higher WF suggests that the electrons need high energy to get detached from the surface while smaller WF suggests that electrons can be easily removed from the surface. This difference implies that electrons on the surface of CVD 1L $MoSe_2$ require less energy to detach compared to those on Exf ML- $MoSe_2$. This WF values are in good agreement with previous theoretical calculation.[7]

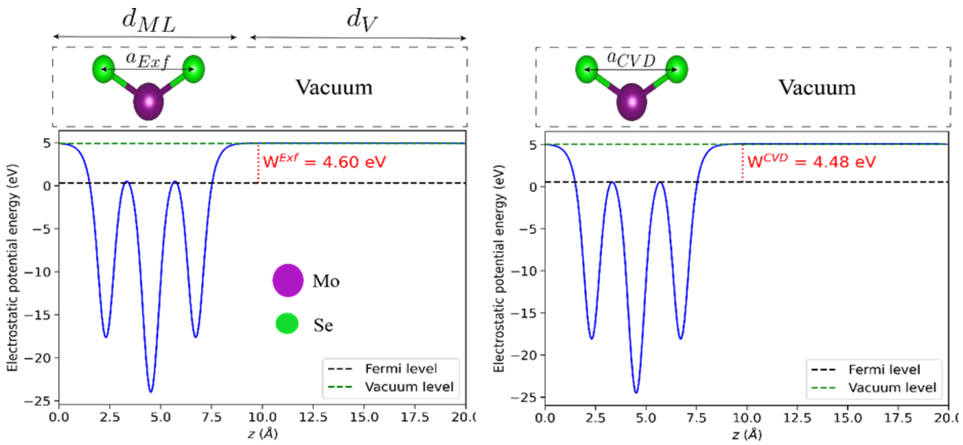

Figure S8: Top panel are the side view of the atomic structure of $MoSe_2$ with lattice parameter a_{Exf} in left and a_{CVD} in right. d_{ML} and d_V are the monolayer and vacuum thickness. The bottom panel are the planar average of the electrostatic potentials (V(z)) distribution of the 1L $MoSe_2$. The values of $W^{CVD/Exf.}$ are indicated. The violet and green balls represent Mo, and Se atoms, respectively.

<u>Reference</u>:

[1] Clark, S. J.; Segall, M. D.; Pickard, C. J.; Hasnip, P. J.; Probert, M. I. J.; Refson, K.; Payne, M. C. First Principles Methods Using CASTEP. 2005, 220 (5–6), 567–570.

[2] Perdew, J. P.; Burke, K.; Ernzerhof, M. Generalized Gradient Approximation Made Simple. Phys. Rev. Lett. 1996, 77 (18), 3865–3868.

[3] Tkatchenko, A.; Scheffler, M. Accurate Molecular Van Der Waals Interactions from Ground-State Electron Density and Free-Atom Reference Data. Phys. Rev. Lett. 2009, 102 (7), 073005.

[4] Giannozzi, P.; Baroni, S.; Bonini, N.; Calandra, M.; Car, R.; Cavazzoni, C.; Ceresoli, D.; Chiarotti, G.L.; Cococcioni, M.; Dabo, I. QUANTUM ESPRESSO: a modular and open-source software project for quantum simulations of materials. J. Phys.: Condens. Matter 21 395502.

[5] Giannozzi, P.; Andreussi, O.; *et al.* Advanced capabilities for materials modelling with Quantum ESPRESSO. J. Phys.: Condens. Matter 29 465901.

[6] Giannozzi, P.; Baseggio, O.; Bonfà, P.; Brunato, D.; Car, R.; Carnimeo, I.; Cavazzoni, C.; de Gironcoli, S.; Delugas, P.; Ferrari Ruffino, F.; Ferretti, A.; Marzari, N.; Timrov, I.; Urru, A.; Baroni, S. Quantum ESPRESSO toward the Exascale. J. Chem. Phys. 2020, 152 (15), 154105.

[7] Han-gyu, K.; Choi, H. J. Thickness dependence of work function, ionization energy, and electron affinity of Mo and W dichalcogenides from DFT and GW calculations. Phys. Rev. B 103, 085404.
